# Viruses of endophytic and pathogenic forest fungi

**DOI:** 10.1007/s11262-020-01763-3

**Published:** 2020-05-09

**Authors:** Abu Bakar Siddique

**Affiliations:** Department of Ecology and Environmental Sciences (EMG), Umeå University, Umeå, Sweden

**Keywords:** Mycovirus, Fungal endophytes, High-throughput sequencing, Fungi, Mycobiome, Endophyte virus

## Abstract

Mycoviruses, just as the fungal endophytes they infect, are ubiquitous biological entities on Earth. Mycoviruses constitute a diverse group of viruses, and metagenomic approaches have—through recent discoveries of been mycoviruses—only recently began to provide evidence of this astonishing diversity. The current review presents (1) various mycoviruses which infect fungal endophytes and forest pathogens, (2) their presumed origins and interactions with fungi, plants and the environment, (3) high-throughput sequencing techniques that can be used to explore the horizontal gene transfer of mycoviruses, and (4) how the hypo- and hypervirulence induced by mycoviral infection is relevant to the biological control of pathogenic fungi.

## Fungal endophytes and mycoviruses

Fungi that reside inside living organisms without causing visible symptoms during at least one part of their life cycle are generally known as endophytic fungi [1, 2]. These fungi are hyperdiverse and ubiquitous, existing in all major habitats—marine, plant, animal, lichen, and soil [3–7]. Therefore, it is not surprising that endophytes perform various functions. For example, mycorrhizal fungi, endophytes, and lichens drive nutrient cycling and influence biomass production through a mutualistic relationship [8]. Leaf, litter and soil fungi include symbiotrophs, saprotrophs, or decomposing fungi which can degrade leaf and litter [7, 9–11]. In grass species, endophytes get shelter, nutrition, and transmission by host propagules; as a favor, endophytes increase host protection from herbivores and increase tolerance against different kind of stress factors, such as drought [12]. In contrast, tree endophytes are horizontally transmitted and predominantly non-systemic in that their life cycles are quite cryptic and they often exert a neutral influence on their host [13].

Endophytes have a wide variety of functions—from mutualistic or symbiotic to pathogenic—that depend on host and/or environmental conditions [14]. For example, endophytes can help a plant become resistant to certain pathogens [15, 16], harmful fungi (e.g., *Melamspora* rust in poplar) and herbivorous insects [17, 18].

**Fig. 1:**
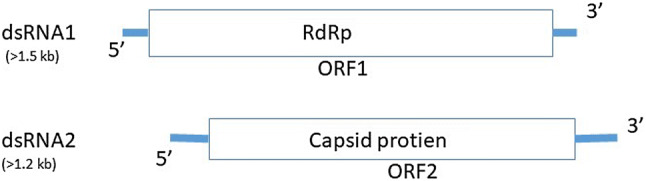
Schematic representation of genome organization of a dsRNA virus. *RdRp* RNA-dependent RNA polymerase, *ORF* open reading frame

Viruses that multiply in fungi are generally known as mycoviruses [19]. The first mycovirus was discovered when the cause for mushroom die-back disease was investigated [20], after which the knowledge base concerning mycoviruses has expanded gradually. At present, hundreds of mycoviruses have been discovered [21], with most of the known species having a doubled-stranded RNA (dsRNA) genome, and a small share having either a single-stranded RNA (ssRNA) and single-stranded DNA (ssDNA) genome. A schematic representation of a dsRNA virus is provided in Fig. 1. According to ICTV (https://talk.ictvonline.org/), mycoviruses are classified into seven families, yet many species are not included in the existing classification system, which is based on genomic structure, virion structure, the amino acid sequence of the RNA-dependent RNA polymerase (RdRp), and the presence or absence of coat protein. The *Totiviridae, Partitiviridae, Megabirnaviridae, Chrysoviridae*, *Quadriviridae* and *Reoviridae* are most abundant mycovirus families [22–25]. Mycoviruses are not considered infectious per se (with a few exceptions), as they lack an extracellular route for infection and spread exclusively via spore production and hyphae conjugation.

The estimated number of fungal endophyte species has constantly increased as environmental sampling and high-throughput sequencing (HTS) technologies have evolved and become cheaper for researchers. Recent estimates suggest that it is likely that between 1.5 and 10 million fungal endophyte species exist [26, 27], and scientists have stated that 30–80% of fungal species may be infected with mycoviruses [23, 25]. These estimates of the sheer number of fungal endophyte species suggests that recent discoveries of novel mycoviruses are just the tip of the iceberg, with most mycoviruses still undiscovered. During the last few years, most mycovirus research has focused on identifying the viruses which infect the pathogenic fungi of important crops, as these discoveries could be relevant to the biological control of harmful fungi. As a result, the mycoviruses which infect non-pathogenic fungi have largely been overlooked. Hence, as most endophytes remain undiscovered due to a presumed lack of pathogenicity, latent forest pathogens could be a great resource for novel mycovirus discovery.

## Origin of mycoviruses

The origin of mycoviruses remains a debated topic, with two main hypotheses dominating the discussion. The first hypothesis for how mycoviruses originated is the ancient coevolution hypothesis, which suggests that viruses and fungi coevolved over time [28]. The second hypothesis proposes that mycoviruses evolved from plant viruses to occupy the niche of infecting plant-associated fungi [19, 25]. This theory cites previous evidence that certain mycoviruses have foreign structural units or domains which are assumed to be of eukaryotic or plant origin [29]. Mycovirus evolution is dominated by strong purifying selection; as such, low genome variability can be expected. Researchers are currently interested in using molecular biology methods to reveal how these structural units affect the host through either viral infection studies or simply expressing the element within fungal cells.

## Viruses of endophytes and forest fungi

Viruses have been detected in all forms of fungi, from endophytes (e.g., *Trichoderma harzianum*) to obligate parasites (e.g., *Puccinia striiformis*) [30, 31]. Bao and Roossinck [32] provided an overview of the putative viruses of 52 distinct endophytes, revealing that almost all endophyte classes are assumed to have mycoviruses living in them (for a more detailed description refer to [32]). A large portion of the currently known mycoviruses infect grass endophytes, with research reporting that 53 different grass endophytes are infected by mycoviruses [33, 34]. More specifically, the dsRNA virus *Epichloe festucae* virus 1 (EfV1) was identified from the grass endophyte *Epichloe festucae* [32]. Tree endophytes can also serve as hosts for mycoviruses. For example, a mycovirus was detected in *Colletotrichum gloeosporioides*, an endophyte which harms cashew trees (*Anacardium occidentale* L.) by causing anthracnose [35]. Furthermore, viruses from the *Endornaviridae* family, which can infect plants, fungi and oomycetes, were found in root mycorrhizal *Ceratobasidium* fungi [36, 37]. Ong et al. studied the fungal symbionts of *Pterostylis sanguinea*, a wild orchid from western Australia, and found 22 novel mycoviruses from the investigated fungi [37]. Moreover, mycoviruses not only infect filamentous, phytopathogenic fungi, but have also been reported in edible mushrooms, which demonstrates their commercial significance [38, 39]. Additionally, the parasitic fungi of mushrooms were found to contain mycoviruses [40].

Mycoviruses are widespread among certain forest fungi. For example, the fungus *Gremmeniella abietina*, which causes canker in coniferous trees across Europe and America, serves as a host for several mycoviruses. Phenotypic changes were frequently observed when the fungus was infected with multiple viruses. Characterizing these viruses, as well as understanding their evolutionary histories, could provide information about their route of transmission and interaction with the host.

**Fig. 2:**
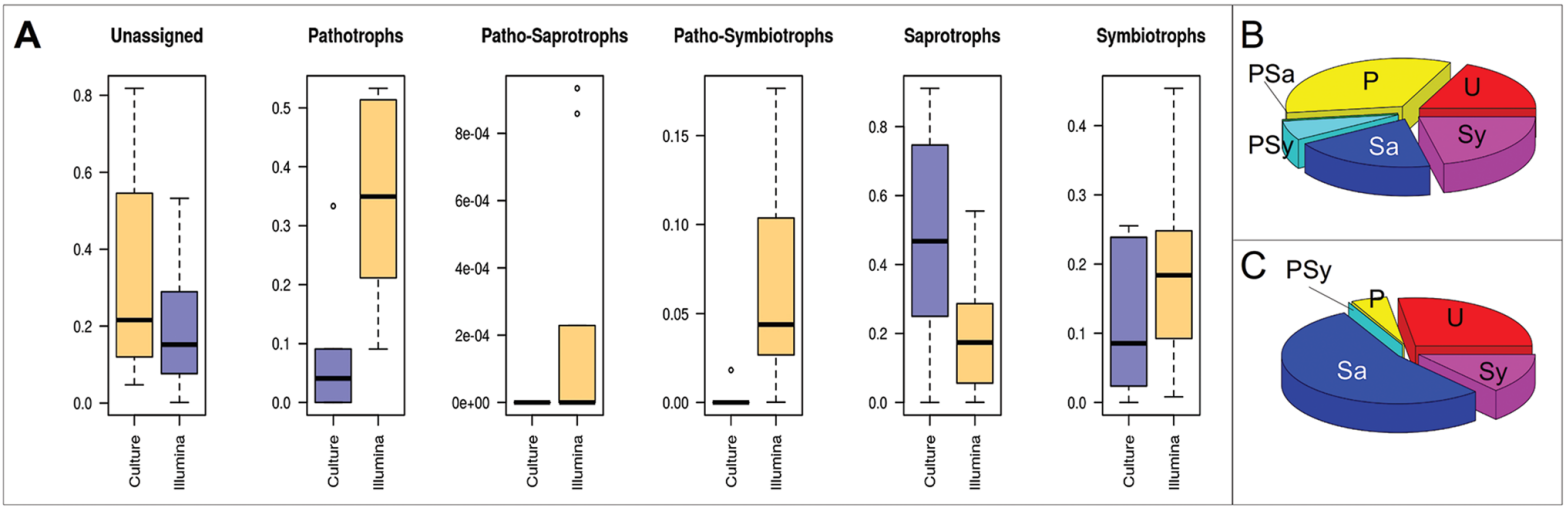
Relative abundance of leaf-inhabiting fungal endophytes of European beech trees among the five main trophic guilds or associations. **a** compares the two methods (Illumina vs cultivation) for each trophic guild and unassigned data. **b** displays the trophic guilds and unassigned taxa for Illumina data, **c** for cultivation data. **b** and **c***U* unassigned, *P* pathotrophs, *PSa* patho-saprotrophs, *PSy* patho-symbiotrophs, *Sa* saprotrophs, *Sy* symbiotrophs, adapted from Siddique et al. [41]

Leaf-inhabiting endophytes—which are part of the mycobiome of a tree—demonstrate various types of trophic associations with their host [41], ranging from pathotrophy to symbiotrophy (Fig. 2). Therefore, it is expected that plant-inhabiting fungal endophytes will reveal diverse new mycoviruses [34, 42]. Another source of novel mycoviruses could be the symptomatic, as well as asymptomatic, fungi associated with woody plants. Notably, research in grapevines revealed 39 viral genomes, of which 38 had not been previously reported [43]. Mycoviral studies are especially relevant when the endophyte hosting the virus confers a plant with protection against herbivorous insects [44].

## Plant-fungi-virus-environment

Plants, fungal endophytes, mycoviruses, and the environment all participate in a four-way interactive system. In natural environments—which are constantly in flux due to various biotic and abiotic factors—these interactions are constantly shifting; for example, abiotic stress such as a drought can favor one of the components, such as the pathogen, and facilitate the infection of the host plant. Moreover, a change in any component of the interactive system may influence the whole system. As a result, a symbiotic effect may disappear while a new relationship arises [45]. It has been speculated that a symbiotic virus can make the host endophyte more, or less, pathogenic through alterations in the phenotype or gene expression profile of the infected endophyte [46]. Previous research has also suggested that these alterations may be heritable, as mycoviruses have been shown to suppress the RNA silencing defense mechanisms of the host, possibly through epigenetic changes [47].

Plants rely on symbiotic relationships with microbes to improve the nutritional condition and manage biotic and/or abiotic stress. Both mutualism and antagonism between plants and endophytes have been extensively studied. Based on environmental conditions, this relationship may continuously shift [48]. Mycoviruses can influence the plant-endophyte relationship by either increasing or reducing host virulence (hyper- and hypovirulence, respectively) or host fitness under altered environmental conditions [32]. On the other hand, as most endophytes do not cause visible symptoms in plants, the mycovirus that exists within a certain endophyte may have a negligible influence on either the endophyte host or the plant which the endophyte is associated with. Nevertheless, several studies have shown that changes in certain fungal traits, e.g., growth of mycelia, sporulation rates, or heat tolerance, following viral infection can lead to either a devastating infestation of the plant or a significantly less harmful fungal infection [34, 49, 50].

## Biological and ecological effects of mycoviruses in endophytic fungi and forest pathogens

Mycoviral infections of fungi exert diverse biological and ecological effects [36]. As previously mentioned, some mycoviruses reduce the virulence of the host fungus (hypovirulence), which can make the fungus less harmful to plants, whereas other mycoviruses have been shown to enhance the virulence of the host fungus (hypervirulence). In this way, depending on the mode of action, mycoviruses could be exploited as potential bioagents to control fungal diseases. This is plausible because mycoviral infections in several fungi have been shown to cause hypovirulence. A well-known example is the use of Cryphonectria hypovirus 1 (CHV1) mycovirus, which reduces the pathogenicity of the fungus *Cryphonectria parasitica,* to curb chestnut blight in America [51]*.* Some additional examples of mycoviruses which cause hypovirulence in the host fungus are Cryphonectria hypovirus 2 (CHV2) in *Cryphonectria parasitica* [52], Sclerotinia sclerotiorum hypovirus 1 in *Sclerotinia sclerotiorum* [53], and W370dsRNA in *Rosellinia necatrix* [54]. Moreover, significant progress has been made in identifying and characterizing the mycoviruses and domains that cause hypovirulence in the host [25, 51, 52]. For example, the vr1 structural domain of Fusarium graminearum virus China 9 (FgV-ch9) was found to cause symptoms in *Fusarium graminearum* [55]. In contrast, several mycoviruses cause hypervirulence following infection [56]. Furthermore, laboratory experiments demonstrated that mycoviral infection of the fungus *B. bassiana* causes a mild hypervirulent effect [57], while another research group found that the presence of viral dsRNA in *Nectria radicicola* increased virulence [58], along with sporulation and laccase activity. In other cases, a mycovirus can enhance certain properties of the host without conferring hypervirulence; for example, the growth rate of *Monilinia fructicola* increased by 10% following infection with three mycoviruses (relative to non-infected cultures) [59]. Moreover, the mold fungus *Sclerotinia sclerotiorum* and white root rot fungus *Rosellinia necatrix* were shown to host many types of mycoviruses that conferred either hypo- or hypervirulence [53, 54, 60–63]. Following mycoviral infection in *Alternaria alternata*, the fungus produced host-specific toxins and caused black spot in Japanese pear [64], while the infection of *Aspergillus fumigatus* by Aspergillus fumigatus tetramycovirus-1 (AfuTmV-1) decreased the survival rate of *Galleria mellonella* larvae [65]. Furthermore, initial evidence suggests that infection by Pseudogymnoascus destructans partitivirus-pa may enhance pigmentation and conidiation in the host fungus, which is responsible for widespread fatality among Northern American bats by causing white-nose syndrome [66].

Virus-fungus interactions can be beneficial, neutral, or harmful for the host [67]. Mycovirus often affect fungal morphology, spore production, growth, virulence, heat tolerance, and toxin production. Perhaps the best known example of virus-fungus interactions is Cryphonectria hypovirus 1 (CHV1)-mediated hypovirulence in *Cryphonectria parasitica,* the fungus which produces lethal cankers on chestnut trees [59, 68]. Moreover, increased virulence and toxin production, as well as irregular growth, were observed in *Alternaria alternata* following mycoviral infection. There is currently no definitive proof about whether fungus-virus interactions help plant hosts survive in extreme environments, with most previous studies focusing on whether endophytes—both infected and non-infected—can confer heat tolerance to plant hosts [33, 69]. For example, the fungus *Curvularia protuberia*, when infected by a mycovirus, helped panic grass (*Dichanthelium lanuginosum*) tolerate excessive heat, and researchers intend to investigate whether this strategy can be extended to tomato plants [70]. Viruses have been shown to alter the transcriptome, small RNAome, proteome, metabolome, lipidome, and epigenome of the host. Transfection protocols allow researchers to study both the host range of a virus and examine host-virus interactions. In this protocol a virus nucleic acid is induced in the same host (genetically same background) and host symptom and defense responses are monitored [52, 71]*.* This interaction could be commercially relevant, as fungi infected by certain mycoviruses may produce secondary metabolites that have potent antimicrobial properties [42]. Hence, a bioreactor system could be developed to extract significant quantities of these antimicrobial compounds from cultured fungal cells.

### Influence on toxin production

Mycoviruses have been found to affect fungal production of mycotoxins, with this effect notable at high temperatures. For example, mycovirus-infected grass fungus *Tolypocladium cylindrosporum* produced less fumonisin B (FB) than the virus-free strain [72], whereas mycovirus-infected *Aspergillus clavatus* isolates produced less patulin than control fungi [73]. Moreover, mycoviral infection in *Fusarium graminearum* has been associated with decreased deoxynivalenol production [74]. In wine yeasts, the synthesis of an allelopathic toxin that inhibits the growth of other fungi increases noticeably upon viral infection [75]. Nevertheless, the mechanisms through which viral infection modifies toxin production remain unclear, and warrant further research.

## Metagenomic studies of fungal endophytes and their mycoviruses

Many different approaches have been used to study the biodiversity of mycoviruses, including enrichment of virus-like particles, extraction of dsRNA, and the HTS of small RNA and total RNA [43, 76, 77]. HTS methods facilitate the rapid discovery of novel viruses by simultaneously generating millions of sequences/reads from hundreds of environmental samples. Furthermore, HTS has the capability to provide high-resolution genomic and community structure data that is crucial for testing new hypotheses [78]. Currently, the most commonly used HTS platforms are Roche 454, IonTorrent, Illumina, PacBio, SOliD and NANOPORE. All of these platforms have distinct drawbacks and benefits based on the type of research they are used for; however, a detailed look into when each specific system should be applied is beyond the scope of this review. It should be stated that older technologies are currently being replaced by superior solutions; for example, most researchers agree that Roche 454 sequencing will soon be antiquated as the Illumina and PacBio systems provide lower error rates at lower prices.

The cultivation-independent analysis of myco- and microbiome communities is commonly referred to as meta'omics, which comprises metagenomic, transcriptomic, proteomic and metabolomic, aspects [11]. Due to the universality of HTS techniques, metagenomic studies have become commonplace in virome research. Current meta'omics studies in the field of virology not only focus on the simultaneous detection, identification, and characterization of novel viruses [79], but are also being performed to gain insight into fungal antiviral defense mechanisms, which may have applications for the biological control of pathogenic fungi.

Advanced mycovirus studies are now focused on providing data on the host transcriptome and RNA signaling [31, 68, 80]. HTS has been successfully used to detect known and unknown viruses from fungal samples [81] as well as detect mycovirus genome segments through small RNA deep sequencing [25, 82]. In addition, host transcriptome and small RNA profiling data have provided insight into the molecular mechanisms underpinning the observed changes in phenotype [83, 84]. Genetic engineering research has started to concentrate on mycovirus-mediated hypo- or hypervirulence [84, 85] due to the clear link between antiviral RNA silencing and mycoviral infection [86]. The fungal antiviral defense is related to small RNA processing. By looking at sRNA accumulation and mRNA signaling, scientists have outlined genetic changes in fungi following mycoviral infection [84], although some research has not found any changes in the levels of mRNA related to gene-silencing proteins following infection [84].

Meta-transcriptomic approaches can also be useful for virus identification [76, 78, 79]. Notably, Gilbert et al. [78] screened transcriptomic data for fungi from the subphylum Pezizomycotina, and found 59 viruses from 44 different fungi based on RNA-seq analyses. As such, environmental sequencing data may prove to be a valuable resource for mycovirus researchers. New mycoviruses could be discovered by analyzing old transcriptomic data which have not been used in virus research before. Furthermore, RNA sequence data in public databases could prove to be an untapped resource for virus discovery [87]. However, researchers should not solely focus on analyzing RNA sequence data, as mycoviruses with a DNA genome have also been discovered. The first instance, a geminivirus-related ssDNA mycovirus which infects, and confers hypovirulence to, *Sclerotinia sclerotiorum,* was reported by Yu et al. [60]. Moreover, cassava associated circular single strand DNA virus was characterized from fungi that target cassava plants [88]. In a rather unique finding, a mycovirus with a DNA genome which was first isolated from a fungal pathogen was found to also infect a mycophagous insect, *Lycoriella ingenua* [89].

## Horizontal gene transfer has revealed foreign structural domains in mycoviruses

During the process of evolution, viruses acquired genetic information from their distant hosts, whether prokaryotes, eukaryotes, plant or animals, by a mechanism known as horizontal gene transfer (HGT). For example, the homologous ‘glycosyltransferase 28’ domain of the *Endornavirus* genus [90] and the ‘S7’ domain of the *Phytoreovirus* genus are widely distributed among plants, bacteria, and fungi [91]. Furthermore, a ‘SMC’ (structural maintenance of chromosomes) domain was discovered in Sodiomyces alkalinus fusarivirus 1 (SaFV1). The SMC domain of SaFV1 are phylogenetically related to the SMC domains of *Fusariviridae* viruses, and distantly related to proteins found in bacterial and eukaryotic organisms [29]. Comparative sequence analyses of unknown viral domains could provide insight into the evolution of mycoviruses, while the role of SMC domains on the infection of fungi—which could be studied through gene expression analyses—could clarify the function of this protein in the virus life cycle. Simpler analyses can be performed by inoculating fungi with mycoviruses which include the SMC domain and following survival rates and/or phenotypic changes. Researchers can determine the function of domains which are postulated to have been acquired via HGT by expressing the protein in fungal cultures and testing whether it has a negative or positive effect on the culture. Overlap extension PCR is a simple and popular technique for cloning a viral domain into a plasmid vector without the need for restrictive endonucleases [92]. Briefly, the prepared plasmid is cloned into *E. coli*, and after protoplast transformation the effects of the studied protein can be monitored through either growth rate, sporulation, or gene expression via qPCR [55, 93]. Phylogenetic analyses can then be used to explore the evolutionary origins of structural and potential HGT-acquired domains present in mycoviruses. Streamlined protocols for horizontal gene transfer, foreign structural domain, and phylogenetic relationship analyses currently exist for mycoviruses (refer to [90, 91]).

Virus metagenomic research has revealed that RNA viruses which infect fungi, plants, and vertebrates evolved by multiple instances of horizontal virus transfer. The analysis of RNA-dependent RNA polymerases (RdRp) and small RNA present in viruses strongly support that various groups of viruses have diverse hosts, including protists, fungi, plants, and animals [94].

Some plant viruses seem to infect fungi and replicate in fungal cells [94–96]. For example, experiments in which fungal spheroplasts were transfected with hop stunt (HSVd), iresine 1, and avocado sunblotch viriods revealed that the tested viruses were able to replicate in at least one of the studied fungi (e.g., *Cryphonectria parasitica, Valsa mali*, and *Fusarium graminearum)* [97]. Furthermore, horizontal transmission of cucumber mosaic virus (CMV) was observed in the phytopathogenic fungus *Rhizoctonia solani* [98]. Far less is known about the mycoviruses that infect, and replicate in, plant cells [99, 100]. Notably, the fungal virus CHV1 was only able to systemically infect *Nicotiana tabacaum* plants when inoculated together with other plant viruses or tobacco mosaic virus (TMV), which indicates facilitative interactions between fungi and plant viruses [101]. Mitoviral sequences have been identified in both fungi and plants, with researchers speculating that ancestral mitoviruses became endosymbionts and, for this reason, partial copies of mitovirus RdRP can be detected in plant nuclear and mitochondrial genomes [67, 102]. Furthermore, protein sequence analyses provide evidence that members of the mycovirus family *Hypoviridae* may be related to plant-infecting Potyviruses [103].

## Biological control strategies for forest pathogens

In the near future, we may see many cases in which biological control—via hypovirulence of pathogenic fungi—is used to curb woody plant diseases. Using mycoviruses to combat woody plant diseases caused by pathogenic fungi is an important research topic because this measure could significantly decrease the use of chemical control, which has adverse environmental impacts. It should be noted that there are already precedents for this type of control strategy, as CHV1 was applied to *Cryphonectria parasitica* in order to combat chestnut blight in America. Martín‐García et al*.* [104] outlined four effective strategies for using mycoviruses to control pathogenic fungi:

i Identify viruses that cause hypovirulence
ii Check if the virus can infect most fungal strains
iii Determine a cost-effective method for disseminating the virus into host populations
iv Test if the virus is persistent in most strains under natural conditions

## Conclusions and future perspectives

This review has presented various viruses of fungal endophytes and forest pathogens and is relevant in the context of fungal research because it has been estimated that up to 80% of fungi are infected by mycoviruses, resulting in positive, negative, or negligible effects in the host. Moreover, the review has covered the biodiversity of mycoviruses, the four-way interactive system including viruses, fungi, plants, and the environment, how mycoviral infection can influence symbiotic relationships, infection-mediated hypo- and hypervirulence, antimicrobial properties, the evolution of viruses through horizontal gene transfer, and the role of mycoviruses disease control strategies.

Impending climate change may support the spread of forest pathogens and diseases and play a role in widespread forest epidemics. Based on significant changes in the environment, alterations in the relationship between endophytic fungi and plants may underlie future outbreaks of fungal infection and disease. This can be expected to increase the chemical control of disease spread, which is worrying as chemical compounds are already excessively used in the fields and forests of many countries, with a clear negative effect on friendly biota [105, 106]. Environment-friendly management, also referred to as biological control or biocontrol, has been touted as a way to eradicate the use chemical fungicides. This review presents various lines of evidence for why researchers should further study the mycoviruses of endophytic fungi to build the knowledge base of virus-endophyte interactions. As our understanding of the viruses of fungal endophytes and forest pathogens is only at the very beginning, it is an ideal time to develop sequenced-based tools for the detection and identification of further mycoviruses. The decreased costs of metagenomics approaches and amount of available bioinformatic approaches mean that researchers can investigate virus genomes in both temporal and spatial scales. Furthermore, the combination of HTS and environment- or biome-wide data could enable researchers to explore mycoviral domains/structures within different endophytes to make evolutionary inferences. Until now, only mycoviruses of widespread pathogenic fungi have been characterized, but this review provides evidence that a diverse set of mycoviruses remain to be discovered from either forest samples or publicly available DNA or RNA sequence repositories.
